# Manipulation of fungal development as source of novel secondary metabolites for biotechnology

**DOI:** 10.1007/s00253-014-5997-8

**Published:** 2014-08-21

**Authors:** Jennifer Gerke, Gerhard H. Braus

**Affiliations:** Institut für Mikrobiologie & Genetik, Georg-August-Universität, Grisebachstr. 8, D-37077 Göttingen, Germany

**Keywords:** Velvet domain proteins, COP9 signalosome CSN, Secondary metabolites, Fungal development

## Abstract

Fungal genomics revealed a large potential of yet-unexplored secondary metabolites, which are not produced during vegetative growth. The discovery of novel bioactive compounds is increasingly gaining importance. The high number of resistances against established antibiotics requires novel drugs to counteract increasing human and animal mortality rates. In addition, growth of plant pathogens has to be controlled to minimize harvest losses. An additional critical issue is the post-harvest production of deleterious mycotoxins. Fungal development and secondary metabolite production are linked processes. Therefore, molecular regulators of development might be suitable to discover new bioactive fungal molecules or to serve as targets to control fungal growth, development, or secondary metabolite production. The fungal impact is relevant as well for our healthcare systems as for agriculture. We propose here to use the knowledge about mutant strains discovered in fungal model systems for a broader application to detect and explore new fungal drugs or toxins. As examples, mutant strains impaired in two conserved eukaryotic regulatory complexes are discussed. The COP9 signalosome (CSN) and the velvet complex act at the interface between development and secondary metabolism. The CSN is a multi-protein complex of up to eight subunits and controls the activation of CULLIN-RING E3 ubiquitin ligases, which mark substrates with ubiquitin chains for protein degradation by the proteasome. The nuclear velvet complex consists of the velvet-domain proteins VeA and VelB and the putative methyltransferase LaeA acting as a global regulator for secondary metabolism. Defects in both complexes disturb fungal development, light perception, and the control of secondary metabolism. The potential biotechnological relevance of these developmental fungal mutant strains for drug discovery, agriculture, food safety, and human healthcare is discussed.

## Introduction

Fungi play an important role in our everyday life, whether positive or negative. On the one hand, they are origins of life-saving and life-enhancing drugs, food additives, and aromas, but, on the other hand, they have the potential to contaminate our crops and food or to lead to serious infections. Fungi, as well as bacteria, plants, and some insects, produce secondary metabolites. These natural products are low-molecular-weight molecules that, unlike primary metabolites, are dispensable for survival of the organism but confer an advantage in specific habitats or during changes in environmental conditions.

**Fig. 1:**
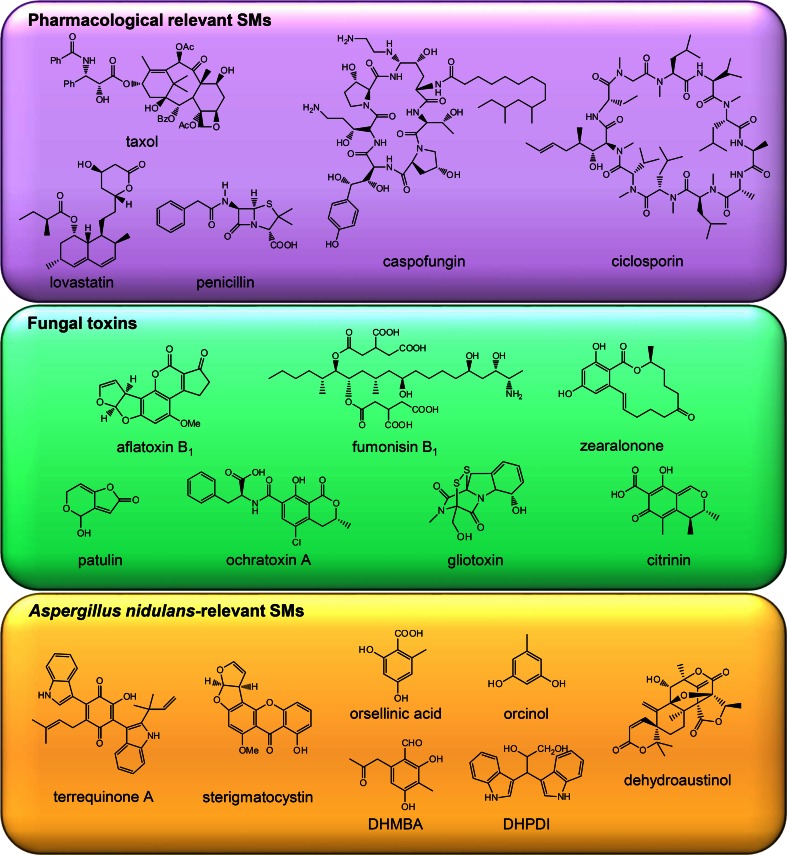
Chemical structures of pharmacological relevant secondary metabolites (SMs), fungal toxins and *A. nidulans*-relevant SMs

Many secondary metabolites possess biological activities that can range from beneficial to harmful (Fig. 1). Beneficial secondary metabolites (SMs) include antibacterial agents such as penicillin, antifungal agents such as caspofungin, anti-cancer drugs such as taxol, immunosuppressive drugs such as ciclosporin, or cholesterol-lowering drugs such as lovastatin. Around 50 % of the newly approved drugs between 1981 and 2010 were of secondary metabolite origin (Newman and Cragg 2012), emphasizing the high importance of studies in this biological field. A growing problem is the tremendous current and future increase in resistances against established antibiotics as was foretold by the WHO (Cooper and Shlaes 2011). The daily application especially of antibiotics in clinical medicine, stock breeding, and agriculture leads to the development of multi-resistances, where known antibiotics are ineffective. Therefore, the discovery of novel drugs is imperative.

The progress in sequencing techniques allows deciphering genomes of more and more organisms within a very short time. The resulting data uncovered a significant genomic potential in many fungi to produce secondary metabolites (Inglis et al. 2013). The biosynthetic genes for the production of SMs in fungi are arranged in clusters. The expression of many of these gene clusters is silenced under standard laboratory conditions. One strategy to discover new bioactive secondary metabolites is the activation of silenced SM gene clusters (Brakhage 2013; Hertweck 2009; Lim et al. 2012). However, the expression of one gene cluster might affect the expression of other gene clusters, what complicates identification of the corresponding SM products (Bergmann et al. 2010; Gerke et al. 2012b; Wiemann et al. 2013).

Fungi are not only producers of drugs, but are also industrially exploited to produce enzymes or food additives. For example, *Aspergillus niger* is used for large-scale fermentation of citric acid and gluconic acid (Berovic and Legisa 2007; Singh and Kumar 2007). In Asian cuisine, *Aspergillus oryzae* is used for fermentation of soybeans, saccharification of rice, and production of alcoholic drinks and rice vinegars (Kobayashi et al. 2007). *Monascus purpureus* is used for natural food coloring (Went 1895). Due to the usage of fungi in food preparation, the knowledge of available secondary metabolite gene clusters becomes even more relevant, as potentially harmful gene clusters might ‘doze’ in the genome and represent an intoxication risk. Prominent examples of deleterious SMs are aflatoxins, a group of mycotoxins produced by several *Aspergillus* species, followed by citrinin and patulin, which are produced by *Aspergillus* and *Penicillium* species, and *Fusarium*-specific toxins such as zearalenone. The contamination of crops with mycotoxin-producing fungi leads to a worldwide yield loss of agricultural crops of more than 10 % (Normile 2010), representing an enormous economic problem. However, fungal spores are not only a risk for plants, but can also pose serious health problems for humans. Inhalation of fungal spores can induce allergic reactions, and in the case of *Aspergillus fumigatus*, *Aspergillus flavus*, and *Aspergillus terreus*, an infection can lead to life-threatening invasive aspergillosis in immunocompromised patients.

A comprehensive understanding of genetic regulation is necessary to compete with nature and to handle the three topics: drug discovery, toxin contamination, and virulence. An interesting approach for biotechnology of yet-unexplored fungi might be to create and use mutations in conserved protein complexes at the interface between fungal development and secondary metabolism. This approach is exemplified here for two well-studied protein complexes: the COP9 signalosome (CSN) and the velvet complex.

## The COP9 signalosome complex CSN: control of fungal protein degradation

### CSN and protein degradation

CSN is a multi-protein complex consisting of up to eight subunits, which is conserved among eukaryotes. It was first identified in plants (COP = constitutively photo morphogenic/de-etiolated/fusca), where mutated *csn* genes induce expression of light-regulated genes in darkness (Wei et al. 1994). In mammals and other higher eukaryotes, disruption of *csn* genes is lethal, whereas deletions in filamentous fungi and unicellular yeasts lead to viable mutants (Busch et al. 2003; Mundt et al. 2002). *Neurospora crassa* harbors only seven subunits, whereas in *Schizosaccharomyces pombe* only six subunits are conserved. The fact that all eight CSN subunits are conserved in *Aspergillus nidulans* (CsnA–CsnH) makes it an attractive model organism to study CSN function (Braus et al. 2010).

**Fig. 2:**
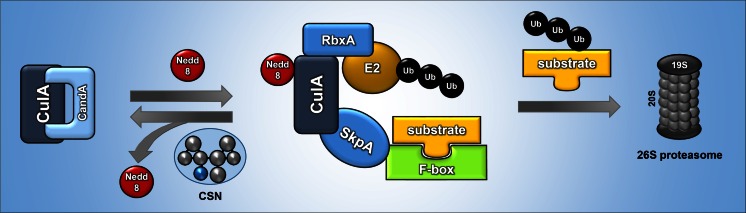
Action of the COP9 signalosome (CSN) complex (in *A. nidulans* nomenclature). An SCF complex represents the largest subclass of CULLIN-RING E3 ubiquitin ligases (CRLs). SCF consists of the subunits SkpA, CulA, an F-box protein and the RING protein RbxA. SCF provides the platform for ubiquitination of substrates. The SCF complex is activated by a neddylation/deneddylation cycle. The CSN complex with its catalytic subunit CsnE deneddylates the SCF complex, which disassembles. The cullin is bound by CandA. A subsequent neddylation step reassembles the active SCF complex. The F-box protein recruits the substrates and RbxA recruits the ubiquitinated E2 enzyme, which then transfers the ubiquitins to the substrates and targets them for degradation by the 26S proteasome

The CSN is structurally similar to the 26S proteasome or the eukaryotic translation initiation factor eIF3, and the eight subunits consist of six PCI (proteasome/COP9/initiation factor) domain proteins and two MPN (Mpr1p and Pad1p N-terminal) domain proteins. The highly conserved fifth subunit CSN5/CsnE harbors the catalytic function of the complex, a JAMM motif with metalloprotease activity, and can recruit the whole CSN complex in the presence of the other subunits (Busch et al. 2003, 2007; Cope et al. 2002). The main function of CSN is to control the activity of CULLIN-RING E3 ubiquitin ligases (CRLs), which ubiquitinate proteins and target them for degradation by the 26S proteasome (Braus et al. 2010; Wei et al. 2008). CRLs are composed of a cullin and a RING-Finger domain protein as a scaffold onto which the ubiquitin-conjugating enzyme E2 binds to transfer the ubiquitin to the substrate (Fig. 2). Additionally, the cullin binds to adaptors that recruit the target substrate. The largest group of CULLIN-RING E3 ubiquitin ligases are SCF (Skp1/cullin-1/F-box) E3 ubiquitin ligases. They consist of a cullin-1 scaffold C-terminally bound to the RING-finger domain protein RBX1/RbxA and N-terminally bound to Skp1/SkpA, which bridges the complex to the substrate-recruiting F-box proteins. The SCF complex is activated by neddylation, which changes the conformation of the complex in such a way that the target substrate and the ubiquitin, which is delivered by the E2-conjugating enzyme through the RING protein, are in sufficiently close proximity (Duda et al. 2008). Neddylation is a process where the small ubiquitin-like modifier Nedd8 is covalently attached to a lysine side chain of a cullin (Wu et al. 2005). This process is reversible by deneddylation. The deconjugation of Nedd8 inactivates cullin complexes. CSN functions as a deneddylating enzyme of cullins. The original finding that in vitro neddylation activates and deneddylation inactivates CULLIN-RING E3 ubiquitin ligases, whereas in vivo CRL activity requires deneddylation, is referred to as the COP9 signalosome paradox (Schmidt et al. 2009). In *A. nidulans*, around 70 F-box proteins are known to be responsible for recruiting different substrates. The activity of the SCF complex is regulated by cycles of disassembly and assembly corresponding to cycles in neddylation and deneddylation. These cycles are presumably necessary to replace F-box proteins which had been ubiquitinated due to the lack of substrates, or to replace F-box proteins which are no more required during developmental processes (Braus et al. 2010). However, CSN is not the only fungal deneddylase. DenA is a second deneddylase, which is conserved in eukaryotes and which physically interacts with CSN. The interaction of both deneddylases affects the half-life of the corresponding proteins (Christmann et al. 2013).

The neddylation and deneddylation cycles of CULLIN-RING E3 ubiquitin ligases require the cullin-associated Nedd8-dissociated protein CandA as an additional control factor (Helmstaedt et al. 2011). CandA stably binds to deneddylated cullins and by this inhibits CRL activity in vitro. In vivo, it was shown that CandA is needed to initiate the exchange of F-box proteins on SCFs (Lo and Hannink 2006). The binding of CandA to the cullin leads to disassembly of the SCF complex, and by a subsequent neddylation step, the complex can be reassembled with an exchanged F-box protein. Taken together, CSN and CandA both activate CULLIN-RING E3 ubiquitin ligases in vivo.

### CSN and fungal development

The general role of CSN in the control of protein turnover results in numerous pleiotropic effects, which have an impact on processes such as hormone signaling, oxidative stress response, DNA repair, cell cycle, growth, differentiation, light signaling, and secondary metabolism (Dohmann et al. 2008; Nahlik et al. 2010; Wei et al. 1994). CSN is only essential in higher eukaryotes, whereas fungal mutant strains of the corresponding genes are viable but often show a disturbed development. In *Saccharomyces cerevisiae*, CSN plays a role in adaptation to pheromone signaling, and *csn* mutants exhibit increased mating efficiency (Maytal-Kivity et al. 2002). In *S. pombe*, *csn* mutants are delayed in progression through S-phase and are more sensitive to DNA damage (Mundt et al. 2002). In the filamentous fungus *N. crassa*, *csn* mutants are impaired in circadian clock control, show a reduced growth rate, and produce less conidia and aerial hyphae than a wild-type strain (He et al. 2005). Similarly, *A. nidulans csn* mutants are blind to light and constitutively induce sexual development even under illumination. However, the sexual development is blocked at the level of primordia, and no mature cleistothecia are formed. In contrast, mutant strains in *denA*, encoding the second deneddylase, which physically interacts with CSN, exhibit an impaired asexual development in *A. nidulans* (Christmann et al. 2013). Therefore, two interacting deneddylases can contribute to two alternative developmental programs in filamentous fungi. A combined transcriptome, proteome, and metabolome analysis revealed that 15 % of genes are mis-regulated during development in Δ*csnE*, a mutant defective in the deneddylase subunit of CSN (Nahlik et al. 2010). The molecular cause of this developmental defect, which is also linked to a mis-regulated secondary metabolism (described in the following chapter), correlates with cellular accumulation of developmentally relevant CULLIN-RING E3 ubiquitin ligases in *csn* mutant strains as a consequence of the dysfunctional neddylation–deneddylation cycle (von Zeska Kress et al. 2012).

### CSN and secondary metabolism

The regulation of CSN controlled processes has tremendous influence on secondary metabolism of an organism. In plants, it was found that the biosyntheses of the hormones gibberellic acid in *Arabidopsis* and jasmonic acid in tomato are reduced in *csn* mutants, leading, for example, to diminished resistance against herbivores and pathogens, whereas the biosynthesis of ascorbic acid (vitamin C) in *Arabidopsis* is elevated (Dohmann et al. 2010; Hind et al. 2011; Wang et al. 2013). In the mold *A. nidulans*, loss of the fifth subunit CsnE, representing the deneddylase subunit, has similar effects on hormone-like psi factor production. The production of the oxylipins 8-hydroxy-9-octadecanoic acid (8-HOE), 8-hydroxy-9,12-octadecadienoic acid (8-HOD), and 10-hydroxy-9,12-octadecadienoic acid (10-HOD) was aberrant, explaining the disturbed sexual development (Nahlik et al. 2010). A defined balance of oxylipins is necessary for correct execution of developmental programs (Tsitsigiannis et al. 2004). Additionally, Δ*csnE* accumulates red pigments that were identified as orcinol and the related phenylethers violaceol I and II, cordyol C, and diorcinol. The same compounds were enriched in the *candA* deletion strains, which further supports the notion that the dysfunctional neddylation–deneddylation cycle of cullin complexes is critical for this phenotype (Helmstaedt et al. 2011).

An untargeted metabolite fingerprinting analysis of Δ*csnE* versus the wild type *A. nidulans* revealed that more than 100 metabolite markers were enriched in the mutant (Nahlik et al. 2010). Additional compounds which normally accumulate in *A. nidulans* are, e.g., precursors of the toxin sterigmatocystin as a member of the aflatoxin polyketide family. In the mutant transcriptome, several gene clusters were upregulated, for which the corresponding products were not yet identified at the time of the analysis. One of them included a gene cluster containing a polyketide synthase (PKS) encoding gene, which is located adjacent to the orsellinic acid gene cluster. The product of the PKS was identified as 2,4-dihydroxy-3-methyl-6-(2-oxopropyl) benzaldehyde (DHMBA, Fig. 1), a polyketide with antibacterial activity (Gerke et al. 2012b). The production of DHMBA illustrated a highly intermingled secondary metabolism. Overexpression of the DHMBA gene cluster abolished the production of another compound, which is only produced by wild-type but not by Δ*csnE* mutant cells. This compound was identified as 3,3-(2,3-dihydroxypropyl) diindole (DHPDI), for which the corresponding biosynthetic genes are not yet identified and presumably located in a different gene cluster of the genome. It is yet unknown how much crosstalk is activated in addition to the mutually exclusive syntheses of either DHPDI or DHMBA. Moreover, the cellular or ecological functions of these metabolites are currently unknown (Gerke et al. 2012b). Further examples of gene clusters that are interdependently regulated are known, and it was shown that these can even be located on different chromosomes (Bergmann et al. 2010; Wiemann et al. 2013).

## Velvet-domain protein complexes: linking transcriptional control and chromatin modification

### Velvet complexes and fungal gene regulation

**Fig. 3:**
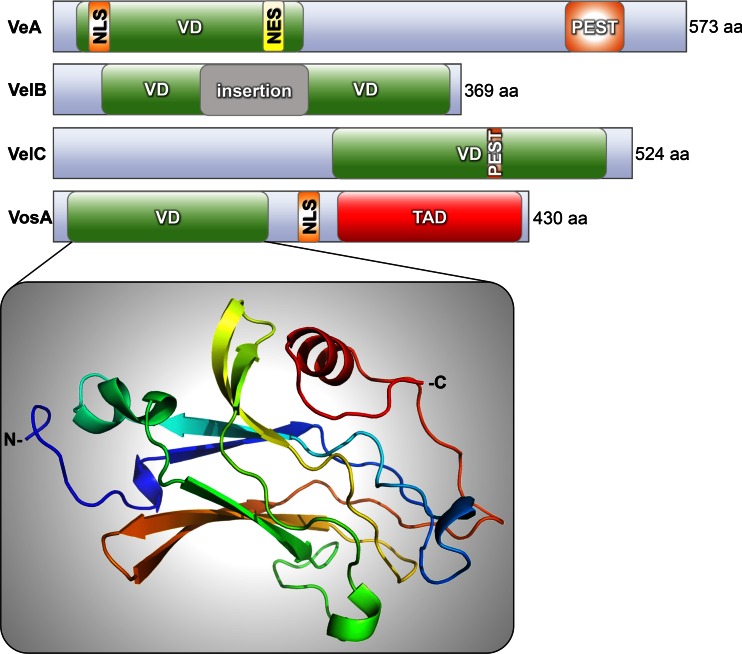
Domain structure of the four velvet-domain proteins and crystal structure of the velvet domain of VosA in *A. nidulans*. *VD*, velvet-domain; *NLS*, nuclear localization signal; *NES*, nuclear export signal; *PEST*, proline- (P), glutamic acid- (E), serine- (S), and threonine-rich (T) region; *TAD*, transcription activation domain

The velvet domain represents a fungus specific protein–protein interaction domain. Only recently, it could be shown that the velvet domain also represents a DNA-binding domain (Ahmed et al. 2013). Velvet-domain proteins are a specific family of regulators, which are conserved in the fungal kingdom from chytrids to basidiomycetes and are particularly well studied in ascomycetes. Velvet proteins are absent in single-cell yeasts as *S. cerevisiae* or *Candida albicans*, which coincides with a lack of secondary metabolite gene clusters in these yeasts (Bayram and Braus 2012; Ni and Yu 2007). The name is based on the first mutant strain for a velvet-domain protein, which had been described in *A. nidulans* as VeA (Velvet A). VeA as the founding member of the velvet family is a light-dependent regulator, which was already introduced to science in 1965 (Kaefer 1965). A mutant strain carried a point mutation in the start codon and produced the truncated version VeA1, missing the first 37 amino acids. The *veA1* strain was used over decades as laboratory strain, because it forms more asexual spores than a wild-type strain with an intact VeA protein. In addition, sexual development is retarded and reduced. VeA harbors the conserved velvet domain in the N-terminal region, as well as a nuclear localization signal (NLS) and a nuclear export signal and shuttles between cytoplasm and nucleus (Fig. 3). Additionally, it contains a C-terminally located proline (P), glutamic acid (E), serine (S), and threonine (T) sequence motif (PEST), which suggests that there is a protein half-life control (Rogers et al. 1986). The velvet domain family consists of four members represented by VeA, VelB, VelC, and VosA of *A. nidulans* (and with similar names in other fungi). All of them carry a velvet domain comprising approximately 200 amino acids (Fig. 3).

VelB (Velvet-like protein B) was the second velvet-domain protein described (Bayram et al. 2008a). An interesting feature is that its velvet domain is interrupted by a 99 amino acids insertion (Ahmed et al. 2013). VosA (viability of spores A), another member of the velvet family, was found during a gain-of-function genetic screen in *A. nidulans* and was described as a high-copy repressor of asexual development which couples spore formation and trehalose biosynthesis (Ni and Yu 2007). Besides an N-terminally located velvet domain, VosA comprises a NLS and a C-terminal transcription activation domain (TAD) (Ni and Yu 2007). VosA is a DNA-binding protein that specifically binds to an 11-nucleotide sequence within the promoter regions of more than 1,500 genes in *A. nidulans* (Ahmed et al. 2013), among them genes involved in asexual development (*brlA*, *wetA*, *vosA*) or trehalose biogenesis (*tpsA*, *treA*). Similarly, the VosA and VelB homologs in *Histoplasma capsulatum* Ryp2 and Ryp3 are DNA-binding proteins (Beyhan et al. 2013). VelC was, until recently, the least studied protein of the velvet family, but, within the last 2 years, it was characterized intensively. It contains one C-terminally located velvet domain with an untypical insertion of a PEST sequence motif which is characteristic for unstable proteins and consists of proline (P), glutamic acid (E), serine (S), and threonine (T) residues (Park et al. 2014).

The 200 amino acids sequence which represents the velvet domain did not reveal significant similarities to other described protein domains. The recently solved crystal structure of the velvet domains of VosA and the heterodimer VosA-VelB demonstrated an unexpected similarity to the Rel homology domain (RHD) of the mammalian transcription factor NF–κB, although the two proteins share only a low amino acid sequence identity of 13.9 % (Ahmed et al. 2013). The family of NF–κB responds to external stimuli. RHD containing proteins control development in mammals, inflammation, and the immune response (O'Dea and Hoffmann 2010; Oeckinghaus et al. 2011). The structural similarity between NF–κB and the velvet domain could be due to independent convergent evolution or due to a common origin for both proteins. If the second hypothesis is true, a common ancestor would have evolved differently, resulting in the control between development and secondary metabolism in fungi and the inflammatory-immune response in mammals (Ahmed et al. 2013).

**Fig. 4:**
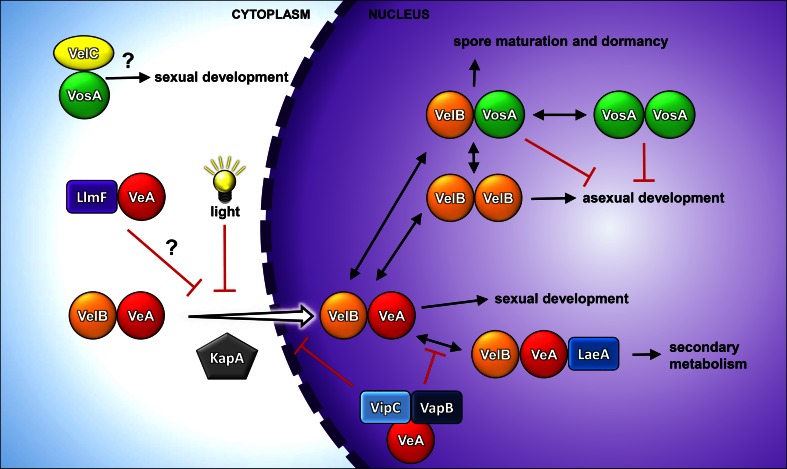
Interplay of different velvet-domain protein complexes linking fungal development and secondary metabolism. During illumination, VeA–VelB are found cytoplasmically. In darkness, VeA–VelB is transported via interaction with KapA into the nucleus, where it initiates sexual development. Via interaction with LaeA, a trimeric complex is formed, which coordinates secondary metabolism. By interaction of VeA with VipC and VapB nuclear import and VelB–VeA–LaeA complex formation is repressed. The VelB–VosA heterodimer is involved in spore maturation and dormancy and represses asexual development. In addition, there are also nuclear VelB and VosA homodimers affecting asexual development. In the cytoplasm, VeA can interact with the methyltransferase LlmF. This complex hinders the nuclear import of VeA. The function of the recently identified cytoplasmic complex VosA–VelC is currently unknown, but it is suggested to be involved in sexual development

The velvet-domain proteins are assumed to be a novel group of transcription factors, and the binding sites for members of the family other than VosA have to be elucidated in the future. The velvet-domain proteins can undergo complex formation among themselves, but also with other proteins, leading to different homo- and heterodimers or heterotrimers (Fig. 4). The formation of these complexes in the cell is assumed to be time- and cell-type specific. VeA can form a complex with VelB. During illumination, VeA is primarily located in the cytoplasm. In the absence of light, VeA containing the NLS is transported into the nucleus via the importin α KapA. VelB contains no NLS itself and is channeled into the nucleus via interaction with VeA–KapA. In the nucleus, KapA dissociates, and the dimeric VeA–VelB complex induces sexual development (Bayram et al. 2008a). The VelB–VelB homodimer is formed in the nucleus, and although it is involved in the regulation of asexual development, its exact function and the DNA-binding specificity is unknown. Besides its interaction with VeA and with itself, VelB can also undergo complex formation with VosA. VosA can also form both a homodimer, which represses asexual development, or the heterodimers VosA–VelB and VosA–VelC (Ahmed et al. 2013; Beyhan et al. 2013; Park et al. 2012b; Sarikaya Bayram et al. 2010). An interaction of VelC with velvet-domain proteins was not only shown in *A. nidulans*, but it was also shown in *Penicillium chrysogenum* and *Fusarium oxysporum* (Kopke et al. 2013; Lopez-Berges et al. 2013). *F. oxysporum* VelC interacts with VeA, and *P. chrysogenum* VelC interacts with VelA and VosA, suggesting the existence of two so far uncharacterized complexes, VelC–VosA and VelC–VeA. Recently, three new VosA interaction partners were found, VoiA, VoiC, and VoiD (VosA-interacting protein), whose function is still unknown (Park et al. 2014).

The first described trimeric velvet complex consists of the two velvet-domain proteins VeA and VelB and the putative methyltransferase LaeA (loss of *a* *flR* expression A). VeA as the central protein is N-terminally associated with VelB and C-terminally with LaeA. The formation of this trimeric complex is light-dependent. The interaction of the nuclear LaeA with the heterotrimeric VeA–VelB in the nucleus links sexual development and secondary metabolism in *A. nidulans* (Bayram et al. 2008a). LaeA contains a SAM- (*S*-adenosylmethionine-) binding site, which is essential for its function, and recently, a novel automethylation mechanism was found (Bok et al. 2006; Patananan et al. 2013). VeA also interacts with two additional methyltransferases VipC (velvet interacting protein C) and VapB (VipC associated protein B). All three methyltransferases LaeA, VipC, and VapB contribute to histone modification and therefore link transcriptional and epigenetic control of gene expression (Sarikaya Bayram et al. 2014). A study on the *A. nidulans* gene encoding the SAM-synthetase, which catalyzes the formation of the main methyl group donor *S*-adenosylmethionine for methyltransferases, also supports an epigenetic link to gene expression in the coordination of fungal development and secondary metabolism (Gerke et al. 2012a).

### Velvet complexes and fungal development

Complete loss of the gene for the light regulator VeA leads to the absence of cleistothecia as closed fruiting bodies for sexual ascospores, whereas the overproduction of VeA results in constitutive cleistothecia formation independent of light conditions (Kim et al. 2002). VeA is therefore a positive regulator of sexual development. In *Aspergillus parasiticus* and *A. flavus*, lack of the VeA counterpart results in a loss of sclerotia formation. Sclerotia are asexual resting structures of these fungi and are reminiscent of cleistothecia with the exception that neither meiosis nor ascospore formation take place (Calvo et al. 2004; Duran et al. 2007). Deletion of the *veA* homolog in the plant pathogens *Fusarium fujikuroi*, *Fusarium graminearum*, and *Fusarium verticillioides* results in a decreased number of (micro-)conidia or an increased macroconidia to microconidia ratio, respectively (Li et al. 2006; Merhej et al. 2012; Myung et al. 2009). Similarly, mutations of the corresponding gene of *N. crassa* impair asexual spore formation (Bayram et al. 2008b). These data corroborate a significant role of VeA in different organisms in development, which can be either asexual or sexual differentiation.

Overexpression of *velB* leads to a twofold increase in the amount of conidia but has no effect on sexual development. Deletion of *velB* in *A. nidulans* leads to a loss of cleistothecia formation. Additionally, the mutant strain shows a reduced number of asexual spores as a result of a downregulation of the asexual marker genes *brlA*, *abaA*, and *vosA*. VelB is part of the two complexes VelB–VosA and VelB–VeA and is therefore involved in the control of both asexual (VelB–VosA) and sexual (VelB–VeA) development (Park et al. 2012b). In the plant pathogen *F. fujikuroi*, loss of the VelB homologs results in similar phenotypes as loss of VeA homologs with decreased amounts of microconidia (Wiemann et al. 2010). In *F. graminearum*, *FgVELB* deletion increases conidiation (Jiang et al. 2012). The spores of a *vosA* or *velB* deletion mutant in *A. nidulans* lack trehalose, rapidly lose cytosol, and have a reduced long-term viability and tolerance to oxidative stress or heat. The VosA–VelB heterodimer represses asexual development by negative feedback regulation of the regulatory gene *brlA* and positively regulates *wetA* and *tpsA*, which are involved in spore maturation and trehalose biosynthesis. Transcription of both, VosA and VelB, is activated by AbaA in phialides.

VelC was characterized in *A. fumigatus*, *A. flavus*, *P. chrysogenum*, *F. oxysporum* and, recently, also in *A. nidulans* (Chang et al. 2013; Kopke et al. 2013; Lopez-Berges et al. 2013; Park et al. 2012a; Park et al. 2014). In *F. oxysporum*, deletion of *velC* leads to an increased amount of microconidia and decreased chromatin accessibility (Lopez-Berges et al. 2013). However, in *A. flavus* and *A. fumigatus*, the deletion has no phenotype. In *A. nidulans*, VelC has an additional role in regulating sexual development (Park et al. 2014). *velC* is expressed during early sexual development, and deletion of *velC* decreases the amount of cleistothecia, whereas overexpression induces cleistothecia production. Therefore, VelC is proposed to be an activator of sexual development. The function of the heterodimeric complex VosA–VelC still has to be elucidated (Park et al. 2014). It was proposed that it acts in cleistothecia formation during sexual development. Additionally, the *velC* deletion increases asexual conidiation, and thus, expression of the asexual regulator genes *brlA*, *abaA*, *wetA*, and *vosA* are upregulated. Due to the fact that the double deletion of *velC* and *vosA* shows the same phenotype as the *vosA* single deletion, it can be assumed that VosA is epistatic to VelC (Park et al. 2014).

The putative methyltransferase LaeA, which links secondary metabolism to development, also has a major impact on fungal differentiation. LaeA is, together with the two velvet-domain proteins VeA and VelB, a member of the trimeric velvet complex. In light, the nuclear LaeA reduces the levels of VosA and VelB and with that favors asexual development, whereas, in the dark, the trimeric complex is formed and initiates sexual development. LaeA controls the switch between asexual and sexual development not only in *A. nidulans* (Bayram et al. 2008a), but also in other ascomycetes like *F. fujikuroi* and *P. chrysogenum* (Hoff et al. 2010; Wiemann et al. 2010). In *A. nidulans*, the *laeA* mutant is reduced in asexual sporulation and produces smaller fruiting bodies compared with the wild type. This is due to the almost complete lack of globose multinuclear Hülle cells, which have been proposed to be required to nurse the fruiting bodies (Bayram et al. 2008a; Sarikaya Bayram et al. 2010). In *A. fumigatus* and *P. chrysogenum*, the conidiophore development is delayed or lacking, respectively (Bok et al. 2005; Hoff et al. 2010). In *A. flavus*, besides aberrant conidiation, no sclerotia are formed (Chang et al. 2012; Kale et al. 2008), and in *F. fujikuroi*, the amount of microconidia is reduced (Wiemann et al. 2010). The two additional methyltransferases VipC (velvet interacting protein C) and VapB (VipC associated protein B), which in addition interact with VeA also have an impact on fungal development in *A. nidulans*. VipC–VapB either act in the nucleus to promote asexual development or are attached to the plasma membrane in a second trimeric complex with the membrane protein VapA which supports sexual development. The molecular mechanism which triggers the dissociation of the two methyltransferases from the plasma membrane is currently unknown (Sarikaya Bayram et al. 2014).

### Velvet complexes and secondary metabolism

A correlation between development and secondary metabolism was already recognized early in fungal research (reviewed in Calvo et al. 2002), and recently, two secondary metabolites (SMs), dehydroaustinol and diorcinol, were found in signaling the induction of sporulation in *A. nidulans* (Rodriguez-Urra et al. 2012), and two SM-synthesizing enzymes of *A. flavus* were found to be essential for proper fruiting body development (Forseth et al. 2013). The velvet-domain protein VeA plays an important role in coordination of development with secondary metabolite production. In addition to its function during fruiting body formation, VeA influences secondary metabolism. A *veA* deletion in *A. nidulans* abolishes sterigmatocystin production, and the mutant produces red pigments instead (Bayram et al. 2008a; Kato et al. 2003). Similarly, in *A. parasiticus* and *A. flavus*, no aflatoxin or aflatoxin precursors are formed in *veA* deletion mutants, and it was shown that *veA* is a global regulator of secondary metabolism (Amare and Keller 2014; Calvo et al. 2004; Duran et al. 2007). Deletion of the *veA* homolog in the plant pathogens *F. fujikuroi*, *F. graminearum*, and *F. verticillioides* disturbs toxin production. The trichothecene production in *F. graminearum* and the fumonisin and fusarin production in *F. verticillioides* are positively affected by Ve1. In contrast, Vel1 of *F. fujikuroi* can as well act as positive (for gibberellins, fumonisins, fusarin C) or negative (for bikaverin) regulator of secondary metabolite production (Merhej et al. 2012; Myung et al. 2009; Wiemann et al. 2010). In the biotechnologically relevant fungi *P. chrysogenum* and *Acremonium chrysogenum*, loss of the VeA homolog results in a reduced production of the antibiotics penicillin and cephalosporin and, in addition, causes defects in development (Dreyer et al. 2007; Hoff et al. 2010).

VelB in *A. nidulans* is not only a developmental regulator, but loss of the corresponding gene also leads to the accumulation of red pigments, similar to the *veA* deletion (Bayram et al. 2008a). In *F. fujikuroi*, loss of the VelB or VeA homologs results in impaired toxin production, and the double deletion of both corresponding genes additionally reduces virulence (Wiemann et al. 2010). In the plant pathogen *F. graminearum*, *FgVELB* deletion disturbs pigmentation and reduces pathogenicity (Jiang et al. 2012). In *P. chrysogenum*, conidiation and the production of penicillin are diminished in a *velC* deletion mutant (Kopke et al. 2013).

The putative methyltransferase LaeA was originally discovered in *A. nidulans* as a suppressor of defects in the gene for the specific transcription factor of aflatoxin and sterigmatocystin cluster genes, AflR (Bok and Keller 2004). LaeA is a global regulator of secondary metabolism and controls, amongst others, the production of prominent natural products such as aflatoxin, sterigmatocystin, terrequinone, lovastatin, penicillin, gliotoxin, fumonisins, and gibberellins (Baba et al. 2012; Bok and Keller 2004; Bouhired et al. 2007; Kale et al. 2008; Seiboth et al. 2012; Wiemann et al. 2010; Wu et al. 2012).

A multicopy suppressor screen to identify proteins able to restore SM production in the *A. nidulans laeA* deletion mutant revealed additional control proteins (Shaaban et al. 2010; Soukup et al. 2012). RsmA (remediation of secondary metabolism A) is a basic leucine zipper transcription factor that was found to bind to the *aflR* promoter and by this to regulate the expression of *aflR* and sterigmatocystin biosynthetic genes (Yin et al. 2012). According to this study, the overexpression of *rsmA* partially restored sterigmatocystin production but not the developmental defects in Δ*laeA* and Δ*veA* (Shaaban et al. 2010). In *A. fumigatus*, the overexpression of *rsmA* increased the production of gliotoxin (Sekonyela et al. 2013).

The second protein found in the multicopy suppressor screen was the histone acetyltransferase EsaA (essential SAS2-related acetyltransferase A), a functional homolog of the *S. cerevisiae* Esa1 (Soukup et al. 2012). The overexpression of *esaA* leads to an increased expression of sterigmatocystin, penicillin, terrequinone, and orsellinic acid gene clusters in *A. nidulans*, but, for full effect, LaeA is required. In contrast to the yeast Esa1, this effect is not restricted to subtelomeric regions. Like for RsmA, the restoration of the *laeA* deletion phenotype by EsaA worked for the secondary metabolism defects, but not for developmental defects.

An additional level of complexity in the regulation of secondary metabolism in fungi is the control of cellular compartmentalization. In a reverse genetics screen to identify LaeA-like methyltransferases, LlmF (LaeA-like methyltransferase F) was identified in *A. nidulans* (Palmer et al. 2013) and shortly followed by Llm1 in *Cochliobolus heterostrophus* (Bi et al. 2013). LlmF is a direct interaction partner of VeA and affects its cellular localization. During overexpression of *llmF*, VeA is primarily found in the cytoplasm, whereas the deletion of *llmF* leads to an increased nuclear localization. By this mechanism, LlmF negatively regulates sterigmatocystin production and, in addition, sexual development (Palmer et al. 2013). Accordingly, in *C. heterostrophus*, Llm1 is a negative regulator of T-toxin production (Bi et al. 2013).

## Putative potential of fungal mutant strains of genes for CSN and velvet complexes in biotechnology

### Drug discovery

We urgently need novel drugs to keep up the pace with the rapid increase of microorganisms that develop resistances against established antibiotics. The greatest inventiveness for new active structures is still provided by nature itself. Fungi with its estimated 1.5 million species worldwide (Hawksworth 1991), each supposed to produce several SMs, constitute a reservoir of so far undiscovered chemical structures, which might serve as matrix for the development of new drugs. A simple approach to activate secondary metabolite gene clusters in fungi, which are silenced during vegetative growth and under laboratory conditions, is to produce and take advantage of mutant strains of the two conserved complexes, CSN and velvet.

As described before, the CSN complex regulates the activation of CULLIN-RING E3 ubiquitin ligases (CRLs) and by this, the protein degradation by the proteasome. Loss of a CSN subunit, especially CSN5/CsnE with its catalytic deneddylase activity, leads to mis-regulation of CRLs and to a disturbed protein degradation. This in turn can lead to stabilization of global or specific transcription factors within SM gene clusters, which, under normal conditions, would be degraded rapidly. By using this approach, around 100 metabolite markers were found to be enriched in a *csnE* mutant in *A. nidulans* (Gerke et al. 2012b; Nahlik et al. 2010). This method can be used to identify new SMs, especially for those with silenced biosynthetic gene clusters or to increase the production of special, already known SMs.

Another possibility to activate secondary metabolite gene clusters is the modification of velvet-complex members. The overexpression of the global regulator encoding gene *laeA* triggers penicillin production in *Penicillium* and additionally lovastatin production in *Aspergillus* (Bok and Keller 2004; Kosalkova et al. 2009). Construction of *laeA* overexpression mutants in different, especially so far metabolically uncharacterized fungi, might lead to the discovery of currently unknown SMs. Furthermore, the formation of the velvet complex is controlled by the subcellular localization of VeA (Palmer et al. 2013). Direct interaction of VeA with the methyltransferase LlmF detains VeA in the cytoplasm and prevents complex formation with VelB and LaeA. In those fungi where this mechanism is conserved, *llmF* deletion mutants should permanently induce secondary metabolism and could consequently be used for the discovery of novel SMs.

Besides LaeA and the other methyltransferases which have been reported here, there are additional, promising methyltransferases such as KMT6 of *F. graminearum*, which are involved in the regulation of secondary metabolism. Loss of KMT6 leads to a lack of H3K27me3 and consequently to a constitutive expression of genes involved in SM biosynthesis (Connolly et al. 2013; Smith et al. 2014).

### Toxin contamination

Whereas the utilization of fungal SMs as drugs positively impacts human health, fungi also produce deleterious mycotoxins that can contaminate our food and cause serious intoxications and diseases. High levels of aflatoxin, one of the most carcinogenic substances known, can trigger aflatoxicosis (also called Turkey X disease), which is characterized by acute hepatic necrosis, liver cirrhosis, and carcinomas. The disease was first observed in England in the 1960s, where thousands of poultry died due to aflatoxin contaminated peanut feed (Forgacs and Carll 1962). During an outbreak in Kenya in 2004, more than 300 people suffered from aflatoxicosis with 125 deaths (Lewis et al. 2005). The most common mycotoxigenic fungi that contaminate crops are aflatoxin- and ochratoxin-producing *Aspergillus* species, ochratoxin- and citrinin-producing *Penicillium* species and fumonisin- and trichothecene-producing *Fusarium* species. The worldwide pre-harvest loss of crops is around 10 % (Normile 2010), whereas the post-harvest loss during storage can be even higher. Commercially, around US$1.2 billion is lost annually due to aflatoxin-contaminated crops (Augusto et al. 2013). Different strategies to reduce toxin contamination and improve food safety have been developed. The most promising approach to control aflatoxin contamination is biological control. The use of non-toxigenic *A. parasiticus* and *A. flavus* strains reduced the pre- and post-harvest contamination of crops by aflatoxins significantly around 70–90 % by competitive exclusion of the wild-type strains in the field (Yin et al. 2008).

A useful starting point to systematically reduce toxicity of fungi is to target global regulators of secondary metabolism, like the conserved regulator LaeA. The deletion of *laeA* was shown to reduce the production of aflatoxin and the aflatoxin-precursor sterigmatocystin in different *Aspergillus* species and the production of trichothecene in *Fusarium* species (Bok and Keller 2004; Crespo-Sempere et al. 2013; Kale et al. 2008; Lopez-Berges et al. 2013). Additionally, loss of the LaeA-interacting protein VeA diminishes the amount of aflatoxin in *Aspergillus* and fumonisins in *Fusarium* (Duran et al. 2007; Myung et al. 2009).

## Virulence

Along with the regulation of secondary metabolism, the proteins involved in velvet complex formation have a tremendous impact on virulence of human and plant pathogenic fungi. The cereal pathogen *F. graminearum* produces a group of mycotoxins called trichothecenes during infection. The deletion of *FgLaeA* or the velvet genes *FgVeA* and *FgVelB* diminishes both trichothecene production and virulence in wheat (Lee et al. 2012; Merhej et al. 2012). In *F. verticillioides*, reduced fumonisins biosynthesis in an *FvVE1* mutant goes along with reduced pathogenicity on maize seedlings (Myung et al. 2009), and in *F. fujikuroi*, single deletions of *Ffvel1*, *Ffvel2*, and *Fflae1* decrease the amount of gibberellic acid and virulence in rice (Wiemann et al. 2010). T-toxin production is correlated with virulence of the maize pathogen *C. heterostrophus*. The loss of ChVel1 and ChLae1 reduces the T-toxin biosynthesis and, as a consequence, also the virulence of the fungus (Wu et al. 2012). The grey-mold disease inducing *Botrytis cinerea* loses pathogenicity when *bcvel1* is deleted, and interestingly, already a single nucleotide polymorphism, leading to a truncated protein not able to enter the nucleus, reduces the virulence on different plants (Schumacher et al. 2012). In *A. parasiticus*, loss of VeA leads to reduced conidiation on peanut seeds (Calvo et al. 2004). Its relative *A. flavus* exhibits a reduced virulence in maize and peanut seeds, when *laeA* or *veA* are deleted (Amaike and Keller 2009). For *F. oxysporum*, it was shown that velvet complex-induced pathogenicity is not only restricted to plants, but is also an important factor in mammals. The opportunistic human and plant pathogen induces systemic fusariosis in immunocompromised patients and can infect about 100 different crops. An important virulence determinant is beauvericin. Deletion of *veA*, *velB*, and *laeA* in *F. oxysporum* reduces beauvericin synthesis and virulence in tomato and in murine model of fusariosis (Lopez-Berges et al. 2013). In *A. fumigatus*, the most common inducer of invasive aspergillosis in mammals, loss of LaeA reduces virulence and the amount of pulmonary gliotoxin in mice (Bok et al. 2005). In the human pathogen *H. capsulatum*, the DNA-binding VosA and VelB homologs Ryp2 and Ryp3 were shown to directly regulate genes involved in pathogenicity (Beyhan et al. 2013). Overall, the velvet complex components, especially LaeA, are promising antifungal drug-targets.

CSN is also an important complex involved in pathogenicity, but, in contrast to the velvet complex, CSN protects from pathogenic attacks. In plants, it was shown that an intact CSN is necessary for biosynthesis of normal levels of jasmonic acid. In *csn*-silenced tomato-plants, the amount of jasmonic acid is reduced, and as a result, the plants are less resistant against herbivores and pathogens (Hind et al. 2011). Consequently, it might be interesting to test whether genetically modified plants with induced *csn* expression could help to reduce fungal contaminations and to improve crop quality.

## Conclusions

Fungi are industrially important producers of drugs and food additives, but they also have a devastating impact on human and animal health and economy. The current state of increasing resistances against established antibiotics, drastic crop losses, and human mortality rates due to fungal contaminations and infections emphasizes the importance of an exhaustive understanding of the underlying genetic mechanisms in fungi. New bioactive SMs need to be discovered, for which the huge fungal kingdom with its numerous members is well suitable. It is equally important that the molecular mechanisms of virulence and toxin production and the control of the biosynthetic pathways will be further illuminated. The CSN and velvet protein complexes act at the interface between secondary metabolism and fungal development. The fact that both complexes are conserved among fungi makes them a perfect target to address issues in agriculture and health, which are highly important for our society. Studies of both complexes in different eukaryotic organisms provided evidence for their conserved functions and showed that both complexes are important players in virulence and production of auxiliary and detrimental SMs.
